# Achieving a Pathologic Complete Response for Locally Advanced Esophageal Adenocarcinoma Using Cone-Beam Computed Tomography-Based Online Adaptive Radiotherapy

**DOI:** 10.7759/cureus.68753

**Published:** 2024-09-05

**Authors:** Nicolas Bachmann, Daniel Schmidhalter, Frédéric Corminboeuf, Ekin Ermis, Daniel M Aebersold, Peter Manser, Hossein Hemmatazad

**Affiliations:** 1 Department of Radiation Oncology, Inselspital, Bern University Hospital and University of Bern, Bern, CHE

**Keywords:** dosimetric parameters, locally advanced esophageal cancer, neo-adjuvant radiochemotherapy, online-adaptive radiotherapy, pathologic complete response

## Abstract

Neo-adjuvant chemoradiotherapy (CRT) and perioperative chemotherapy are different strategies for treating non-metastatic esophageal cancer (EC). The advantages of neo-adjuvant therapies are primarily seen in patients who achieve a pathologic complete response (pCR) and therefore show higher survival rates and better prognosis. In general, less than one-third of patients with EC experience pCR after neo-adjuvant therapies; however, patients with esophageal adenocarcinoma (AC) demonstrate lower rates of pCR compared to those with esophageal squamous cell carcinoma (SCC), respectively. Herein, we describe two cases of locally advanced esophageal AC treated with cone-beam computed tomography (CBCT)-based online adaptive radiotherapy (ART) on the ETHOS platform. Both patients received CRT with 50.4 Gy in 28 fractions, combined with weekly carboplatin and paclitaxel. For each fraction, we evaluated scheduled and adapted plans using dose-volume histogram (DVH) data, and patients were treated with the superior plan. We prioritized ensuring optimal coverage of the planning target volume (PTV) over limiting the dose to organs at risk (OARs) when selecting the superior treatment plan. In this instance, we present the translation of superior dosimetric data into clinical benefits, as evidenced by an excellent pathologic response.

## Introduction

Esophageal cancer (EC) ranks as the seventh most prevalent cancer globally and the sixth highest contributor to cancer-related deaths [1]. The standard treatment for EC involves preoperative chemoradiotherapy (CRT) or perioperative CRT combined with surgery in the neo-adjuvant setting, and definitive CRT in cases where surgery is not feasible. Despite advancements in oncological outcomes achieved through multimodal treatments, as opposed to surgery alone, the five-year survival rate for patients with EC remains low, with less than 20% [2]. Given that a pathologic complete response (pCR) is associated with improved oncological outcomes [3], optimizing neo-adjuvant therapy is crucial for achieving higher rates of pCR. Maximal coverage of the planning target volume (PTV), along with a reduced dose to organs at risk (OARs), are the main goals for optimizing neo-adjuvant CRT. Esophageal motion related to directional shifts, dilatations proximal to the tumor due to esophageal stenosis, tumor shrinkage, and various stomach positions are the main obstacles to achieving optimal PTV coverage during the course of CRT. Adaptive radiotherapy (ART) offers the possibility of daily re-planning and, therefore, could overcome these obstacles. Herein, we describe the use of cone-beam computed tomography (CBCT)-based online ART for two cases of locally advanced esophageal adenocarcinoma (AC).

## Case presentation

Case 1 presentation

Baseline Patient Information

An 80-year-old male patient with a histopathologically confirmed diagnosis of locally advanced esophageal AC was referred to the Department of Radiation Oncology after presentation at a multi-disciplinary upper gastrointestinal tumor board. During the initial presentation at our clinic, the patient reported experiencing retrosternal pressure for the past couple of weeks. However, he denied experiencing dysphagia and did not exhibit any weight loss. The patient's performance status was very good, with an Eastern Cooperative Oncology Group (ECOG) score of 0. The tumor was located in the gastro-esophageal junction, beginning 30 cm from the incisors, over a distance of 8 cm, and was classified as Siewert type I.

Case 2 presentation

Baseline Patient Information

A 74-year-old male patient with a histopathologically confirmed diagnosis of locally advanced esophageal AC was referred to the Department of Radiation Oncology after a presentation at a multi-disciplinary tumor board. During the initial presentation at our clinic, the patient presented with progressive dysphagia (Common Terminology Criteria for Adverse Events (CTCAE) grade II) that had developed over several months, along with a weight loss of 10 kg. Despite these symptoms, the patient's performance status was good, with an ECOG score of 1. The tumor was located in the gastro-esophageal junction, beginning 37 cm from the incisors, over a distance of 10 cm, with extensive infiltration of the stomach and regional lymph nodes, and was classified as Siewert type II.

**Figure 1:**
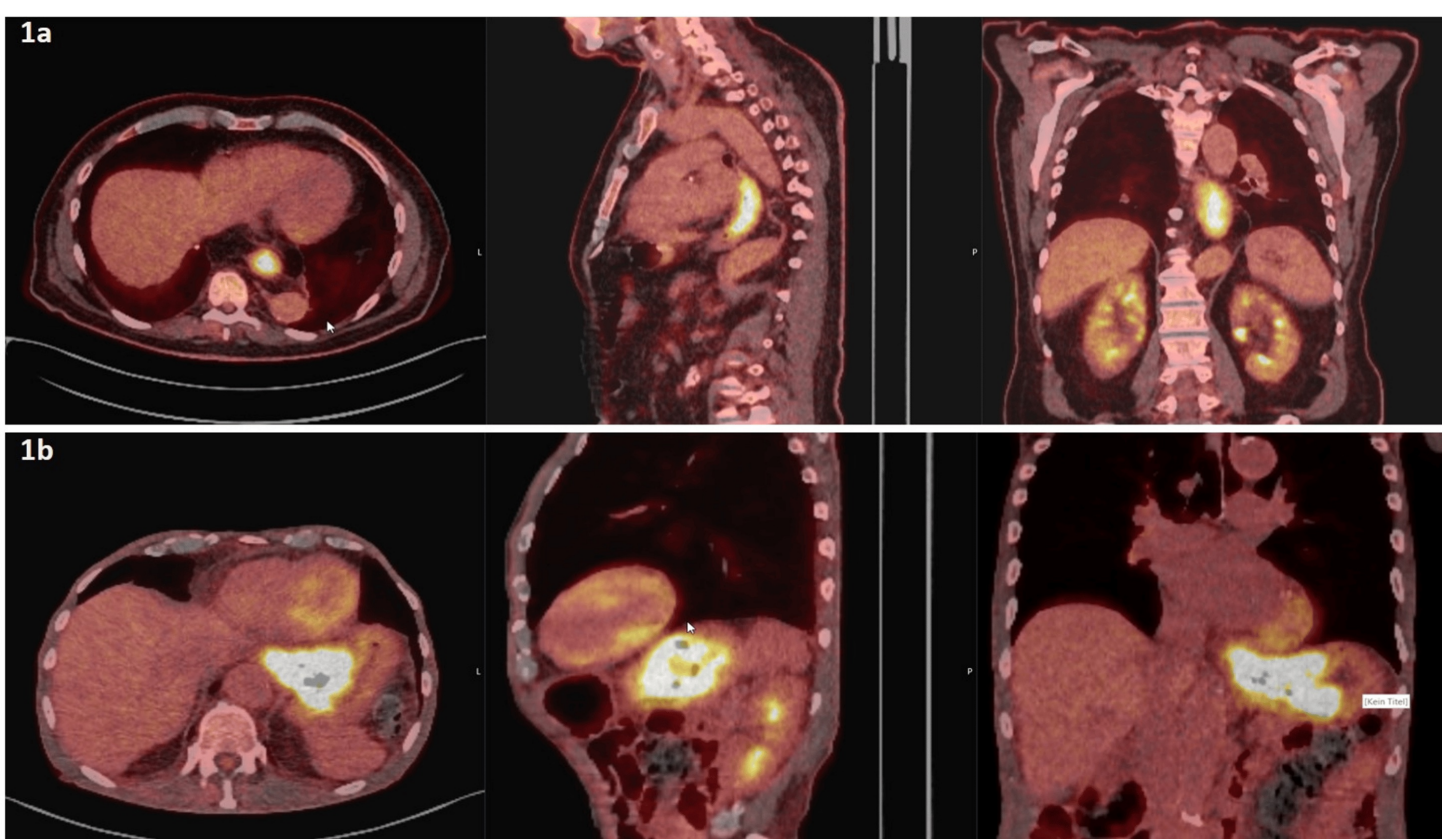
Primary tumor location and extent on FDG-PET/CT for Patient 1 (1a) and Patient 2 (1b) FDG-PET/CT: Fluorodeoxyglucose-positron emission tomography/computed tomography

With suspicion of diaphragm infiltration on positron emission tomography/computed tomography (PET/CT) images, Case 1’s TNM classification was cT4a N0 M0 (Figure 1a). The nodal status was assessed using PET/CT and endoscopic ultrasound (EUS). With suspicion of diaphragm infiltration on PET/CT examination, the tumor in Case 2 had the following TNM classification: cT4a N2 M0 (Figure 1b). Here, the nodal status was assessed using PET/CT and EUS, similar to Case 1.

Treatment planning and delivery

**Figure 2:**
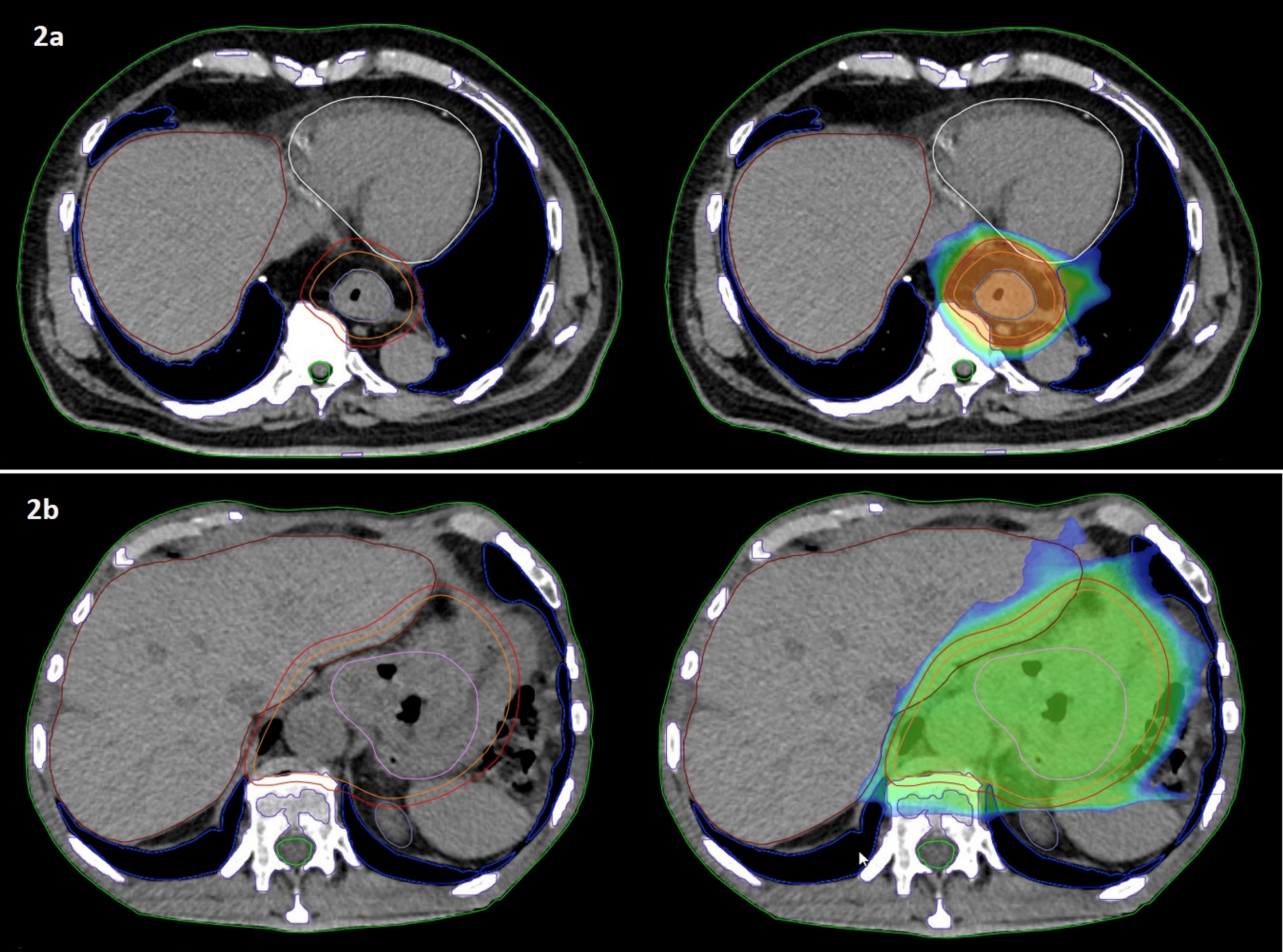
Target volume contours (CTV, orange; PTV, red) on reference plans for Patient 1 (2a) and Patient 2 (2b) CTV: Clinical target volume; PTV: Planning target volume

The patients received neo-adjuvant treatment with 50.4 Gy in 28 fractions using online ART, combined with five to six cycles of weekly carboplatin/paclitaxel. For generating the reference RT plan, we performed a free-breathing 3D planning CT. The patient lay down on a custom immobilization device (VacFix) in a supine position with both arms overhead. We instructed our patients to fast for at least four hours before the planning CT and before every RT fraction to avoid significant changes in stomach position. We imported PET/CT images to the ETHOS treatment planning system (TPS) and registered them to the plan CT using rigid registration. Target volume delineation was done according to international guidelines [4] for EC, and the PTV was generated with a 5-mm expansion of the clinical target volume (CTV) using the derived structure function. Dose constraints for OARs, including the lungs, heart, kidneys, liver, and spinal cord, are listed in Table 1. We set the priority for PTV coverage and dose constraints of the heart and lungs as the most relevant OARs. RT was delivered in both cases with a 12-field intensity-modulated RT (IMRT) plan (Figure 2).

**Table 1:** Metrics/constraints required to be met for radiotherapy (RT) planning PTV: Planning target volume; CTV: Clinical target volume; OARs: Organs at risk

Target volume	Priority	Planning goal	Acceptable variation
PTV	1	D99% ≥ 99%	D99% ≥ 95%
	1	D1% ≥ 101%	D1% ≥ 105%
	3	D95% ≥ 100%	D95% ≥ 98%
CTV	3	D99% ≥ 100%	D99% ≥ 98%
	3	D1% ≥ 101%	D1% ≥ 105%
	3	D95% ≥ 100%	D95% ≥ 98%
OARs	-	Constraints	-
Heart	2	Dmean ≤ 17 Gy	Dmean ≤ 18 Gy
	3	V40 Gy ≤ 78%	V40 Gy ≤ 80%
Lungs	2	V20 Gy ≤ 18%	V20 Gy ≤ 20%
	3	V5 Gy ≤ 78%	V5 Gy ≤ 80%
	3	Dmean ≤ 9 Gy	Dmean ≤ 10 Gy
Liver	2	V30 Gy ≤ 35%	V30 Gy ≤ 40%
	2	Dmean ≤ 17 Gy	Dmean ≤ 18 Gy
Kidneys	2	Dmean ≤ 9 Gy	Dmean ≤ 10 Gy
Spinal cord	2	Dmax ≤ 40 Gy	Dmax ≤ 45 Gy

Online ART workflow: 1) CBCT acquisition: We chose a “thorax-fast” protocol to reduce artifacts. 2) Influencers review: The esophagus, heart, lungs, and stomach are the influencers for the “lower esophagus” template. The influencers help the artificial intelligence (AI)-based system to shape and position daily target volumes appropriately. 3) Target volumes review: gross tumor volume (GTV) and CTV could be evaluated and adjusted based on physician decision. Finally, the PTV was derived automatically from the CTV. 4) Plan selection and online quality assurance (QA): Based on a synthetic CT, two RT plans are available to choose from: “scheduled” and “adapted” plans. The scheduled plan is a recalculation of the reference RT plan on the synthetic CT, while the adapted plan is a new optimization of the reference plan on daily anatomy with initial constraints. For the adapted plan, our medical physics team did a daily independent dose calculation with the aid of Mobius3D version 4.0.2 (Varian, Erlangen, Germany) during every session. 5) Second CBCT and dose delivery: Directly before dose delivery, we performed another CBCT to check for possible movements of the patient and adjusted his position accordingly.

Data collection, dosimetric analysis, and clinical outcomes

**Figure 3:**
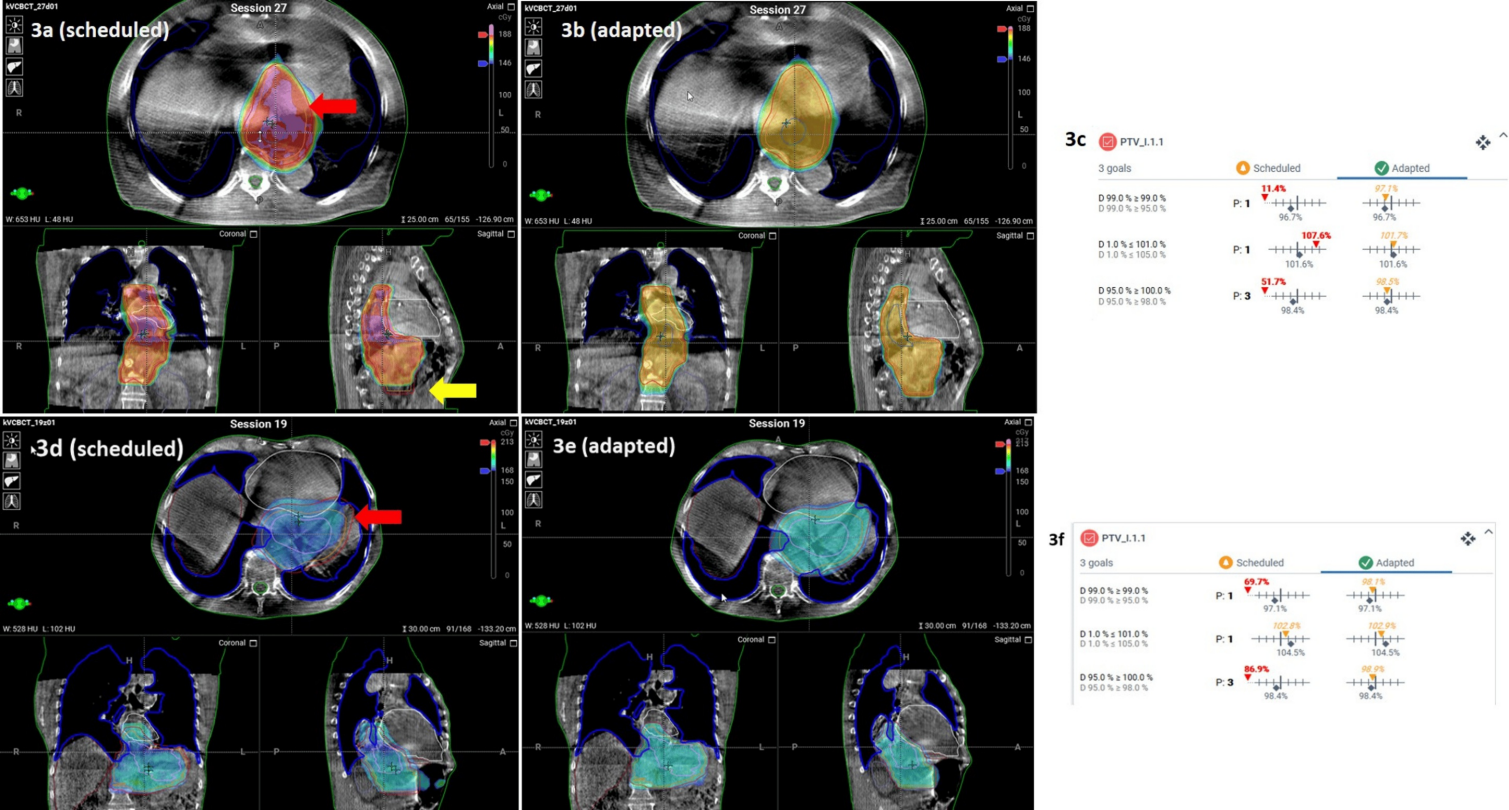
Comparison of PTV coverage between scheduled and adapted plans for Patient 1 and Patient 2, highlighting areas of hotspots and under-coverage Patient 1: Comparison of PTV coverage between scheduled (3a) and adapted (3b) plans for a single fraction. Areas of hotspots (red arrow) and PTV under-coverage (yellow arrow) by scheduled plan. Values from DVH for PTV coverage (3c). Patient 2: Similarly, PTV coverage for scheduled (3d) and adapted (3e) plans for a single fraction. The area of PTV under-coverage is marked with a red arrow. Values from DVH for PTV coverage (3f). PTV: Planning target volume; DVH: Dose-volume histogram

A logbook was established to record the data from each online ART session. Considering all 28 fractions for each case, PTV-D99% (minimum dose) and PTV-D95% (coverage) improved significantly with adapted plans by 22% and 3.5% (Case 1, p < 0.001), as well as 10% and 2.5% (Case 2, p < 0.001), respectively, compared to scheduled plans (Figures 3a-3f).

**Figure 4:**
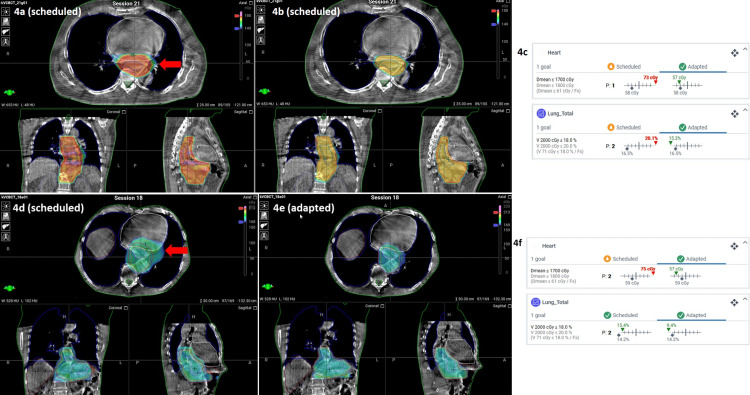
Comparison of radiation dose to the heart and lungs between scheduled and adapted plans for Patient 1 and Patient 2, with areas of excessive dose marked by red arrows Patient 1: Comparison between scheduled plan (4a) and adapted plan (4b) for radiation dose to the heart and to the lungs by a single fraction. Areas of excessive dose are marked with a red arrow. Values from DVH for the dose to OARs (4c). Patient 2: Comparison between scheduled plan (4d) and adapted plan (4e) for radiation dose to the heart and to the lungs by a single fraction. Areas of excessive dose are marked with a red arrow. Values from DVH for dose to OARs (4f). DVH: Dose-volume histogram; OARs: Organs at risk

Furthermore, ART lowered the mean heart dose by 18% and 12%, and lung V20 Gy by 10.5% and 29%, significantly (p < 0.001) for each case, respectively (Figures 4a-4f).

The therapy was well tolerated by both patients. Following the completion of CRT, Patient 1 reported experiencing dysphagia (CTCAE grade I), accompanied by a loss of appetite and a weight loss of around 4 kg. Due to an extensive maculopapular itchy rash, we omitted the last cycle of chemotherapy. Patient 2 experienced an improvement in dysphagia (CTCAE grade I) at the end of CRT, without loss of appetite or weight. Four weeks after the completion of neo-adjuvant CRT, both patients presented themselves in our clinic with no signs of radiation-induced acute toxicity. After six and three months of CRT, respectively, patients received minimally invasive McKeown esophagectomy with extended two-field lymphadenectomy. The pathology report confirmed pCR with TNM classification as follows for both cases: ypT0 ypN0 L0 V0 Pn0 R0. 

## Discussion

In this report, we describe two cases of locally advanced esophageal AC treated with online ART and CRT in the neo-adjuvant setting. We achieved excellent outcomes, with pCR in both cases and maintained an acceptable level of toxicity.

Patients with EC, who are classified as non-responders to neo-adjuvant CRT, show significantly worse oncological outcomes, even in comparison to patients with upfront surgery [5]. In a review and meta-analysis, Gaber et al. demonstrated 22% and 32% pooled prevalence of pCR after neo-adjuvant CRT for AC and squamous cell carcinoma (SCC), respectively [3]. Considering that AC is the most common histological subtype in Western countries, there is room for improvement in neo-adjuvant therapy in patients with EC.

Total neo-adjuvant therapy (TNT) delivers most of the oncological treatments before surgery and has shown promising results, with high rates of pCR for rectal cancer [6]. Until now, the data regarding TNT for esophageal AC is sparse; however, it could show promising results, with improved disease-free survival (DFS), overall survival (OS), and high rates of treatment completion [7]. The latter is an important factor, as, for example, the ESOPEC trial, using perioperative chemotherapy with fluorouracil, leucovorin, oxaliplatin, and docetaxel (FLOT), showed low rates of adjuvant treatment completion, with only 52.5% (American Society of Clinical Oncology (ASCO) 2024). Most of the studies with the TNT concept for esophageal AC used induction chemotherapy followed by CRT in their regimens, achieving pCR rates between 19% and 35% [7]. Furthermore, there are several ongoing prospective trials evaluating the role of TNT for EC. For example, the RACE trial, a prospective randomized phase 3 study, will compare perioperative chemotherapy (FLOT regimen) versus induction chemotherapy (two cycles of FLOT) followed by CRT [8]. Implementing TNT could lead us to non-surgical management of EC; therefore, achieving a complete response is essential for this approach. As we know from the preSANO trial, adequate clinical response evaluation after neo-adjuvant CRT for EC could be achieved with EUS, biopsies, and fine-needle aspiration of suspicious lymph nodes [9]. If we hypothesize that high rates of pCR could be achieved by appropriate regimens and sequencing of CRT, optimal coverage of target volumes is crucial for this goal. The inter-fractional variability in tumor position and shape can be significant, necessitating the use of large PTV margins to adequately cover the esophageal tumor [10]. These variations in tumor position between fractions can ideally be accomplished through daily online adaptation and re-planning [10]. In an in-silico study, Boekhoff et al. showed the potential of online MR-guided radiotherapy (MRgRT) not only to reduce radiation dose to the heart and lungs but also to target tumors more precisely [11]. Using online ART could open new perspectives to implement a wait-and-watch strategy and avoid surgery in patients with EC, especially within the scope of the TNT concept.

## Conclusions

Herein, we have described two cases of esophageal AC treated with CBCT-based online ART. Despite locally advanced tumors with infiltration of adjacent organs and regional lymph nodes, pCR could be achieved after neo-adjuvant CRT. We conclude that online ART not only could improve target volume coverage but also spare better OARs, and therefore plays a role in therapy optimization for patients with esophageal AC.
